# Tissue Engineered Skin Substitutes Created by Laser-Assisted Bioprinting Form Skin-Like Structures in the Dorsal Skin Fold Chamber in Mice

**DOI:** 10.1371/journal.pone.0057741

**Published:** 2013-03-04

**Authors:** Stefanie Michael, Heiko Sorg, Claas-Tido Peck, Lothar Koch, Andrea Deiwick, Boris Chichkov, Peter M. Vogt, Kerstin Reimers

**Affiliations:** 1 Department of Plastic, Hand- and Reconstructive Surgery, Hannover Medical School, Hannover, Germany; 2 Laser Zentrum Hannover e.V., Hannover, Germany; University of Tennessee, United States of America

## Abstract

Tissue engineering plays an important role in the production of skin equivalents for the therapy of chronic and especially burn wounds. Actually, there exists no (cellularized) skin equivalent which might be able to satisfactorily mimic native skin. Here, we utilized a laser-assisted bioprinting (LaBP) technique to create a fully cellularized skin substitute. The unique feature of LaBP is the possibility to position different cell types in an exact three-dimensional (3D) spatial pattern. For the creation of the skin substitutes, we positioned fibroblasts and keratinocytes on top of a stabilizing matrix (Matriderm®). These skin constructs were subsequently tested *in vivo,* employing the dorsal skin fold chamber in nude mice. The transplants were placed into full-thickness skin wounds and were fully connected to the surrounding tissue when explanted after 11 days. The printed keratinocytes formed a multi-layered epidermis with beginning differentiation and *stratum corneum*. Proliferation of the keratinocytes was mainly detected in the suprabasal layers. *In vitro* controls, which were cultivated at the air-liquid-interface, also exhibited proliferative cells, but they were rather located in the whole epidermis. E-cadherin as a hint for adherens junctions and therefore tissue formation could be found in the epidermis *in vivo* as well as *in vitro*. In both conditions, the printed fibroblasts partly stayed on top of the underlying Matriderm® where they produced collagen, while part of them migrated into the Matriderm®. In the mice, some blood vessels could be found to grow from the wound bed and the wound edges in direction of the printed cells. In conclusion, we could show the successful 3D printing of a cell construct *via* LaBP and the subsequent tissue formation *in vivo*. These findings represent the prerequisite for the creation of a complex tissue like skin, consisting of different cell types in an intricate 3D pattern.

## Introduction

Major burn injuries often prove difficult in therapy due to their complexity, the high risk of infection, the large area which might be affected and the potential destruction of deeper skin layers including the dermis. Often, the availability of autologous split-thickness skin grafts and keratinocytes for wound coverage is limited, especially in case of large burned areas. Therefore, the need of skin substitutes for temporary or permanent wound coverage is high. Several skin substitutes like Integra® and Matriderm® are already employed in the clinical application, being complemented by the use of autologous split-thickness skin grafts [1]–[3]. While Integra® serves to prepare the wound bed in preparation for transplantation with autologous split-thickness skin three weeks later, Matriderm® is used in a single step procedure and must be covered immediately. Nevertheless, full success in burn wound regeneration has not been reached yet, neither under functional nor under aesthetic aspects. In nearly every case of treating large and deep burn injuries discolouring or scarring remains, the latter leading to undesirable contractions. Also, neither hair follicles nor sebaceous and perspiratory glands can be regenerated.

Tissue engineering promises to have high potential in the production of new skin. In this context, it remains a challenge to create a precise and complex new tissue comprising several cell types which are arranged in a specific 3D pattern. Furthermore, the different tissue functions strongly depend on its specific structure and on the cells which are influenced by their distinct microenvironment [4]. For example, the formation of vessels in a skin equivalent cultivated *in vitro* is thought to be dependent on the direct interaction of endothelial cells with fibroblasts and their secreted extracellular matrix proteins and growth factors [5]. Bioreactors are used for the *in vitro* cultivation of complex tissues offering the possibility to mimic and control the desired microenvironment [6].

One solution for the problem of creating complex 3D tissues might be the use of LaBP. It offers the possibility to produce specific high resolution two-dimensional (2D) as well as 3D patterns, incorporating different cell types like human osteosarcoma and mouse endothelial cells [7], human osteoprogenitor cells [8], rodent olfactory ensheathing cells [9], human endothelial cells [10] and human adipose derived mesenchymal stem cells which can subsequently be differentiated to fat [11] as well as bone and cartilage [12]. Cells – including rat Schwann and astroglial cells, pig lens epithelial cells [13], Chinese hamster ovarian cells, human osteoblasts [14], murine embryonal carcinoma cells [15], and fibroblasts and kerationcytes [16] – survive the transfer without damage and alteration of cell phenotype. This represents a major prerequisite for the use of LaBP in tissue engineering. Commonly, also the terms cell printing or simply bioprinting are used. In advance of the *in vivo* testing of the here produced skin substitutes we could already show tissue formation and functional cell-cell contacts in corresponding 3D tissue constructs *in vitro* [17].

In this study, *via* the use of LaBP, we created a multi-layered, fully cellularized skin equivalent for the future treatment of burn patients. The transplanted skin equivalent was tested *in vivo* for its ability to form tissue as well as cellular behaviour of the printed cells, the differentiation of the keratinocytes and potential neovascularisation using the dorsal skin fold chamber in mice. *In vitro* controls supplemented the *in vivo* experiments.

## Materials and Methods

### Cell Culture

NIH3T3 fibroblasts (DSMZ, Braunschweig, Germany) and HaCaT keratinocytes (DKFZ, Heidelberg, Germany) have previously been labelled by stable transduction with lentiviral or gammaretroviral vectors encoding for either eGFP or mCherry [17]. In the following the four resulting cell lines are named accordingly: NIH3T3-eGFP, NIH3T3-mCherry, HaCaT-eGFP and HaCaT-mCherry. Fibroblasts were cultivated in Dulbecco’s modified Eagle’s medium (DMEM) with high glucose (4.5 g/L) (PAA, Pasching, Austria) supplemented with 10% fetal bovine serum (FBS) (Biochrom, Berlin, Germany), 1% of 100 mM sodium pyruvate (Biochrom), and 1% of penicillin/streptomycin (Biochrom) whereas keratinocytes were grown with DMEM/Ham’s F12 medium (PAA) supplemented with 10% FBS and 1% of penicillin/streptomycin.

### Cell Transfer and Production of the Transplant

Cells were arranged in 3D skin constructs using LaBP (as previously described in [16] [18]–[19]). Briefly, the setup consists of two co-planar glass slides. The upper one is coated with a thin layer of laser absorbing material (here 60 nm of gold) and a layer of biomaterial to be transferred (here 60 µm of cell containing collagen). This glass slide is mounted upside-down above a second (receiver) glass slide. The laser pulses are focused through the upper glass slide into the laser absorbing layer, which is evaporated locally. The vapor pressure propels a small amount of the subjacent biomaterial towards the receiver glass slide. By moving the glass slides relative to each other, arbitrary patterns of biomaterial can be produced. By repeating this procedure layer-by-layer also 3D patterns can be generated.

For the here presented skin substitutes the cells were trypsinized and centrifuged at 400 g. A pellet containing 1.5 million cells was resuspended in a mixture of 37 µl collagen (Collagen Type I, Rat Tail, BD Biosciences, Bedford, MA, USA), 5 µl phosphate buffered saline (10× PBS, Biochrom) and 0.85±0.5 µl sodium hydroxide (1N NaOH, Sigma-Aldrich) for neutralization (pH = 7.1±0.3) prior to the transfer. For the skin substitutes 20 layers of fibroblast-containing collagen and 20 layers of keratinocyte-containing collagen were printed subsequently onto a sheet of Matriderm® (2.3 cm×2.3 cm, Dr. Suwelack Skin & Health Care, Billerbeck, Germay), used as a stabilization matrix.

The printed cells were kept in the incubator under submerged conditions over night. The next day (defined as day 0), nine round pieces (diameter 6.0 mm) were removed from the large construct with a biopsy punch, three of which were implanted into the skin fold chambers *in vivo* (one per mouse). As four independent printing processes were conducted, altogether 12 animals were used. The remaining six pieces of each printing process served as *in vitro* controls. Two of them were directly fixed on day 0 to depict the situation at the beginning of the experiments, whereas the remaining four pieces were raised to the air-liquid-interface. *In vitro* controls were then fixed on days 5 and 11 (duplicates per time point) and *in vivo* specimen on day 11.

### Cultivation of Constructs in vitro

Constructs were raised to the air-liquid-interface and cultivated with differentiation medium on top of plastic platforms. The latter consisted of cell strainers (BD Biosciences, Bedford, MA, USA) turned upside down. The medium was composed of DMEM high glucose medium (PAA) mixed with the same amount of DMEM/Ham’s F12 medium (PAA), supplemented with 1% FBS, 1% penicillin/streptomycin, 10^−7^ mM isoprenaline hydrochloride (Sigma-Aldrich, Steinheim, Germany), 10^−7^ mM hydrocortisone (Sigma-Aldrich) and 10^−7^ mM insulin (insulin bovine pancreas, Sigma-Aldrich). The resulting calcium chloride concentration of the culture medium is 158.3 mg/L.

### Animals and Ethics Statement

All animal experiments were evaluated and approved by the responsible animal care committee (Nds. Landesamt für Verbraucherschutz und Lebensmittelsicherheit) and the Hannover Medical School (Institut für Versuchstierkunde). The animals (male BALB/c-Nude mice, 8 weeks) were purchased from Charles River and kept in the local animal care facility according to the institution guidelines. They received standardized food and water, living with a day-night cycle of 12 hours each. Animals were used for the experiments when they were at least 12 weeks old.

### Skin Fold Chamber

**Figure 1:**
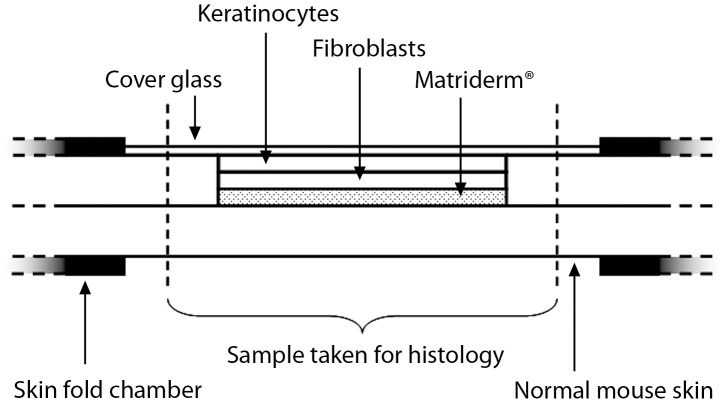
Scheme of the utilised dorsal skin fold chambers in mice. The chambers are attached to the back skin of the mice. The printed skin construct consisting of 20 layers of fibroblasts and 20 layers of keratinocytes on top of Matriderm® is placed into a round full-thickness wound in the mouse skin, while the opposite side remains intact. To close the chamber a cover glass is used.

The dorsal skin fold chamber was used for the evaluation of tissue engineered skin *in vivo* as published previously [20]. The skin constructs were placed into full-thickness wounds, while the skin on the other side of the chamber remained intact. All surgery was performed under isoflurane anesthesia, and all efforts were made to minimize suffering. The mice were sacrificed on day 11 after implantation, preparing the constructs surrounded by normal skin for histological analyses (Figure 1). Analogous constructs - without cells - have already been assessed *in vivo* [20] and are used as a comparison in this study.

### Histology

Samples were fixed in 4% paraformaldehyde, embedded in paraffin and subsequently cut to sections of 5 µm thickness. Masson’s trichrome stainings were conducted following standard procedures.

### Immunohistochemistry

In order to detect the presence of e-cadherin, collagen IV, cytokeratin 14 and Ki67, immunohistochemistry was carried out. For Ki67 (Thermo Fisher, RM9106_S0, 1∶200) and e-cadherin staining (Santa Cruz, SC 7870, 1∶300) the deparaffinised and rehydrated paraffin sections were incubated in a 99°C heated water bath for 25 min (Ki67) and 15 min (e-cadherin), respectively, before blocking. For cytokeratin 14 (Biozol, DBB-DB099-1, 1∶300) and collagen IV (Abcam, Ab6586, 1∶2000) stainings the sections were incubated in a 37°C warm water bath for 10 min, using 100 ml 0.2 N HCl solution with 100 mg pepsin. All sections were blocked with 2% FBS in PBS at room temperature for 30 min, followed by incubation with first antibody in 1% FBS in PBS at 4°C over night. After washing with PBS for all but the collagen IV staining a goat anti rabbit secondary antibody (Sigma-Aldrich, A3687, 1∶1000) coupled to an alkaline phosphatase was employed. The samples were incubated at 37°C for 1 h. As a substrate nitro-blue tetrazolium-5-bromo-4-chloro-3'-indolyphosphate (Roche, Mannheim, Germany) was used. In case of the collagen IV primary antibody, a biotin coupled secondary anti rabbit antibody (Dako, E0432, 1∶400) was used for 90 min at room temperature. Subsequently, the Vectastain ABC Kit (Vector Laboratories, Peroxidase Standard PK-4000) was employed for 60 min at room temperature to enhance the signal. The latter was then visualised with diaminobenzidine tetrahydro chloride (ICN98068). Staining of all antibodies was detected with a light microscope (Olympus).

## Results

### Operations and Macroscopic Evaluation

To evaluate skin constructs *in vivo* generated *via* LaBP, we printed 20 layers of a keratinocyte cell line (HaCaT) on top of 20 layers of a fibroblast cell line (NIH3T3) by an established laser-assisted bioprinting procedure. Using stable transduction, cell lines were labelled with genes encoding for green or red fluorescent proteins, respectively: NIH3T3-eGFP, NIH3T3-mCherry, HaCaT-eGFP and HaCaT-mCherry. Matriderm® was used as a carrier matrix to enhance stability of the constructs for transplantation (Figure 1). The skin constructs were placed into full-thickness skin wounds in the dorsal skin fold chamber preparation in mice in such a way that the constructs and the surrounding skin laid in close contact to each other. Uninjured skin from the opposite side of the back fold served as a control for all experiments. Analogous constructs without the cells [20] serve as a comparison to this study.

**Figure 2:**
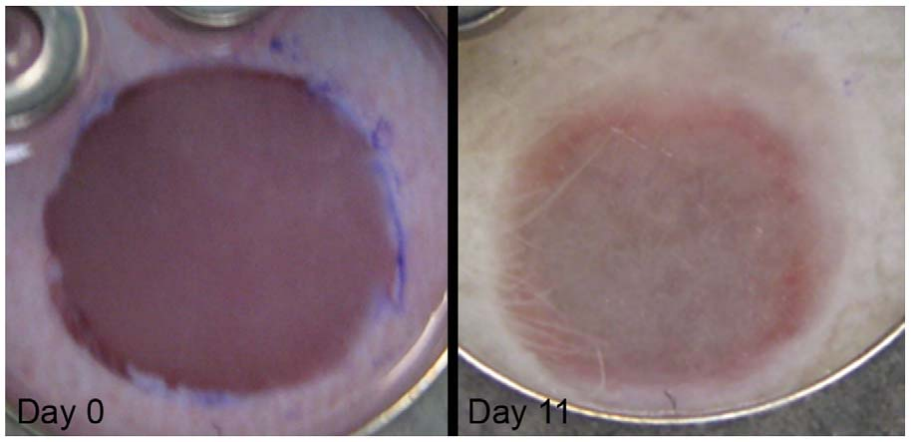
Tissue engineered skin construct in the dorsal skin fold chamber in nude mice. The pictures show a skin construct inserted into the wound directly after the implantation (left) and on day 11 (right). The implanted constructs were created *via* LaBP, consisting of 20 layers of fibroblasts and 20 layers of keratinocytes on top of Matriderm®^.^ They fill the full-thickness wound completely.

All animals survived the surgical intervention and implantation procedure and tolerated the chambers well, showing no signs of discomfort or changes in sleeping and feeding habits. After 11 days, the borders of the skin constructs and the surrounding mouse skin were tightly grown together so that no sharp linings were visible between the two tissue types any more (Figure 2). During the course of time, the previously shining surface of the constructs became matt. Neither inflammatory/necrotic processes nor contraction of the wounds could be detected.

### Formation of Skin-like Tissue in vivo by Printed Skin Constructs

**Figure 3:**
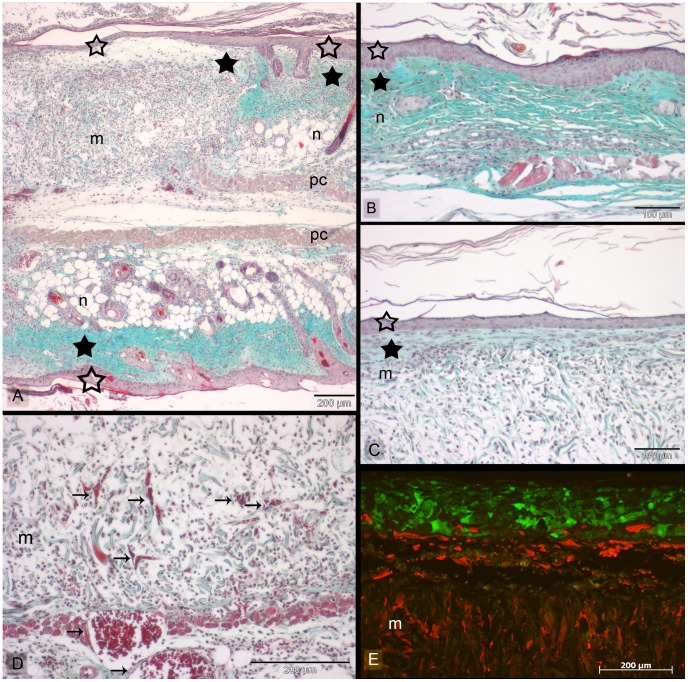
Histological sections of the tissue engineered skin constructs *in vivo.* Skin constructs were implanted in dorsal skin fold chambers in mice for 11 days. Sections were stained with Masson’s trichrome (A–D) or analyzed with fluorescence microscopy (E), respectively. (A) illustrates an overview with the junction between the inserted skin construct (m = Matriderm®) and native mouse skin (n) at the wound edge after 11 days in the dorsal skin fold chamber in mice. The intact mouse skin opposite of the skin construct can be seen in the lower part of the picture (n) (see also Figure 1). The skin construct and the intact skin part in the sandwiched skin are separated by the *panniculus carnosus (pc)*. Both in native mouse skin (B) and the printed skin construct (C) a dense epidermis (empty asterisks) and a corneal layer can be observed. In case of the skin construct, the epidermis is formed by the printed keratinocytes (E). This can clearly be seen by the green fluorescence emitted by the used HaCaT-eGFP cells. The fibroblasts (NIH3T3-mCherry) partly migrated into the Matriderm® (yellowish fibres). The fibroblasts, which stayed on top of the Matriderm®, display an outstretched morphology (C), being accompanied by collagen deposition (filled asterisks). Blood vessels (arrows) can be detected in the skin constructs (D). Scale bars depict 200 µm (A, D, E) and 100 µm (B, C).

**Figure 4:**
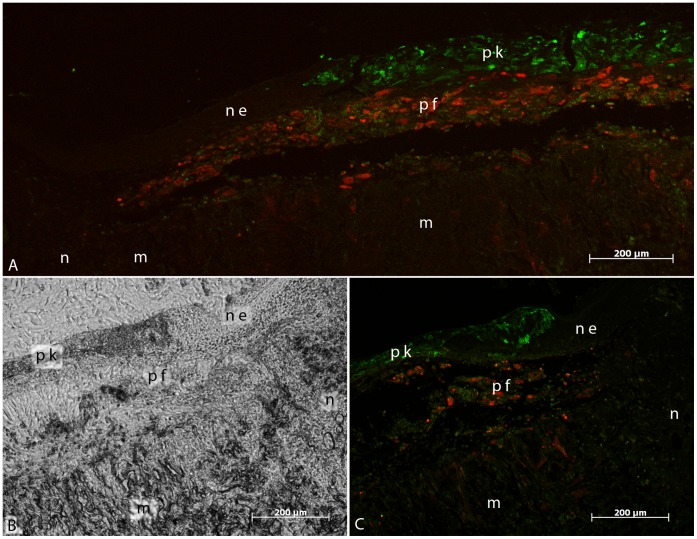
Fluorescent pictures of the tissue engineered skin constructs *in vivo*. Skin constructs were implanted in dorsal skin fold chambers in mice for 11. The sections show an overview of the junction between the inserted skin construct (m = Matriderm®) and native mouse skin (n) with either fluorescence microscopy (A, C) or transmitted light microscopy (B). Two different situations concerning the epidermis were observed during analysis of the junction zones: In some cases, as depicted in (A), the normal mouse epidermis (ne) started to grow on top of the Matriderm®, where it connected to the epidermis formed by the printed keratinocytes (pk). The latter were labelled in green by stable transduction (HaCaT-eGFP). In other cases, as depicted in (B) and (C), the epidermis formed by the printed keratinocytes ended at the border of the Matriderm®, synchronous to the presence of the printed fibroblasts (pf) labelled in red (NIH3T3-mCherry). As can be seen by comparing (B) and (C) - which depict the same location - the keratinocytes even partly grew on top of the normal mouse epidermis. In both cases, the printed fibroblasts formed a multi-layer tissue underneath the printed keratinocytes. Partly, they also migrated into the Matriderm®. All scale bars depict 200 µm.

First of all, the survival and tissue formation of the printed skin cells was of particular interest. On top of the fibroblasts and the Matriderm®, the keratinocytes (HaCaT-eGFP) developed a dense stratified tissue (Figure 3E), similar to normal epidermis as can be seen in the Masson’s trichrome staining (Figure 3A, C). In some samples, this was followed by a corneal layer (Figure 3A, C). However, the epidermis in the skin constructs was less thick than in native mouse skin. Besides, no rete ridges could be found in the skin constructs. After 11 days the tissue developed by the printed cells was connected to the surrounding native mouse skin tissue at the wound edges. Neither an interruption of the epidermis nor a gap between the dermis and the Matriderm® could be observed at the junction between the skin constructs and the mouse skin (Figure 3A, Figure 4). In this context, two different situations could be observed: In some cases, the normal mouse epidermis started to grow on top of the Matriderm®, becoming connected to the epidermis which was formed by the printed keratinocytes (Figure 4A). In other cases, the epidermis formed by the printed keratinocytes ended simultaneously with the printed fibroblasts (NIH3T3-mCherry), directly at the border of the Matriderm®. Partly, the keratinocytes even grew on top of the normal mouse epidermis (Figure 4B, C).

Furthermore, the migration pattern of the printed fibroblasts was assessed. As can be seen in the histological sections, the fibroblasts partly migrated into the Matriderm®, closely following the fibres of the latter (Figure 3E, Figure 4). Some fibroblasts remained on top of the Matriderm®, composing a multi-layer sheet of tissue (Figure 3C) and secreting collagen as can be seen in the trichrome staining. As such, the printed skin cells survived well and formed a multi-layer, keratinized skin equivalent.

**Figure 5:**
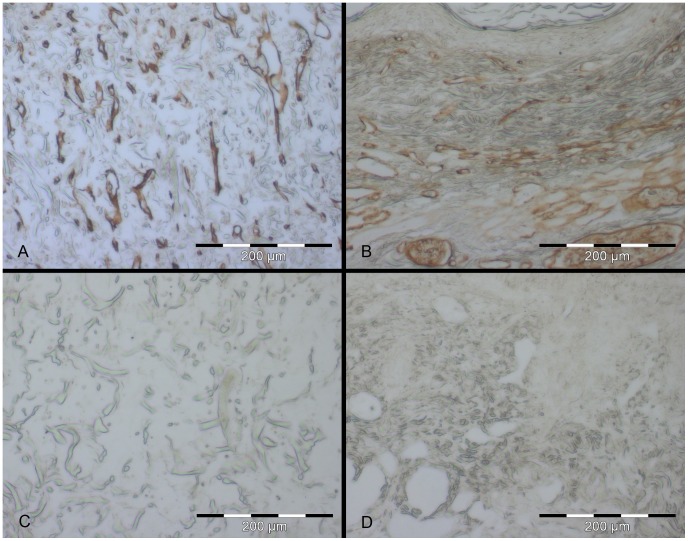
Blood vessel detection *via* immunohistochemistry in skin constructs cultivated *in vivo* for 11 days. Skin constructs were cultivated *in vivo* for 11 days in the dorsal skin fold chamber in mice. Collagen IV expression (brown) – indicating blood vessels/capillaries – can be detected in the Matriderm® as small tubes reaching from the wound bed in the direction of the cells (A). Small and large blood vessels are present in the normal mouse skin (B). Matriderm® (C) and normal mouse skin (D) without first antibody serve as the respective negative controls. Scale bars depict 200 µm each.

One important issue in respect of the use of skin substitutes is their vascularisation. Here, small blood vessels could be found in the skin constructs which seem to grow in from the depth of the wound bed as well as from wound edges into the Matriderm® in the direction of the printed cell layers (Figures 3D and 5).

**Figure 6:**
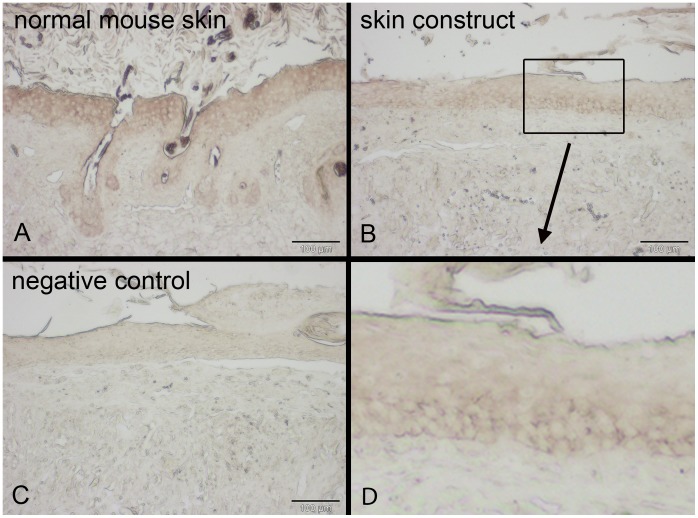
E-cadherin detection *via* immunohistochemistry in skin constructs cultivated *in* *vivo* for 11 days. Skin constructs were cultivated *in vivo* for 11 days in the dorsal skin fold chamber in mice. E-cadherin expression (dark brown) can be found throughout the epidermis in both normal mouse skin (A) and the skin constructs (B). Normal mouse skin without first antibody serves as a negative control (C). All scale bars depict 100 µm.

Adherens junctions – containing especially e-cadherin - are essential for stable cell-cell contact and can abundantly be found in epithelia like skin. Therefore, e-cadherin can be used as a hint for epithelia formation and consequently for tissue development. By means of immunostaining, e-cadherin could be detected between the keratinocytes of the skin constructs inserted into the wounds. Here, the pattern of the e-cadherin localisation is the same as in native skin and can be found in the whole epidermis (Figure 6).

**Figure 7:**
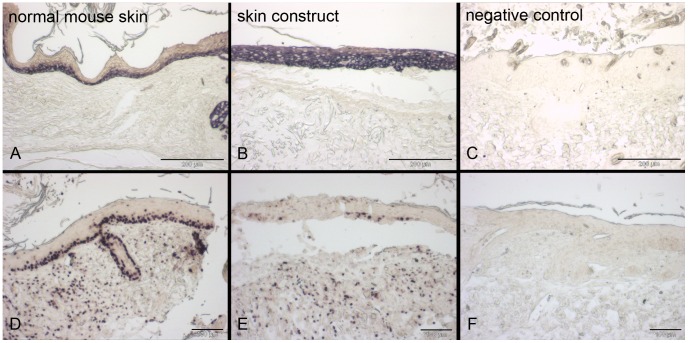
Detection of cytokeratin 14 and proliferation *via* immunohistochemistry in skin constructs cultivated *in* *vivo*. Skin constructs were cultivated *in vivo* in the dorsal skin fold chamber in mice for 11 days. The left column shows normal mouse skin, the middle column the skin construct and the right column the respective negative controls (normal mouse skin without first antibody) of the immunohistochemistry stainings. Cytokeratin 14 expression is limited to the suprabasal layers of the epidermis in mouse skin (A) but present in the whole epidermis in the skin constructs (B). Proliferation *via* Ki67 can be detected in the suprabasal layers of the epidermis and in the dermis in both normal mouse skin and skin constructs (D, E). Scale bars depict 200 µm (A–C) and 100 µm (D–F).

One important characteristic of an epidermis is the differentiation of the keratinocytes. Cytokeratin 14 is a marker for undifferentiated keratinocytes. The corresponding immunostaining revealed the presence of cytokeratin 14 in the whole epidermis of the skin constructs (Figure 7A–C). In native skin cytokeratin 14 staining could be found in only the suprabasal layers.

In contrast, Ki67 as a proliferation marker, showed a signal mainly in the suprabasal layer of the skin constructs (Figure 7D–F), indicating that only those keratinocytes maintained their proliferating state. Note that in the skin constructs only a few cells showed a positive signal for Ki67 whereas in the normal mouse skin nearly all suprabasal cells were stained. Fibroblasts in the dermis showed proliferation in the skin constructs as well as in the native mouse skin.

### In vitro Controls of Printed Skin Constructs

As a control, printed skin constructs were also cultivated *in vitro* with the addition of differentiation medium. Samples were taken for histology on day 0-at the same time point as the corresponding transplants were inserted into the chambers of the mice – to demonstrate the starting conditions of the experiments. Further on, samples were also secured on day 11, corresponding to the end of the *in vivo* experiments and in between (on day 5).

**Figure 8:**
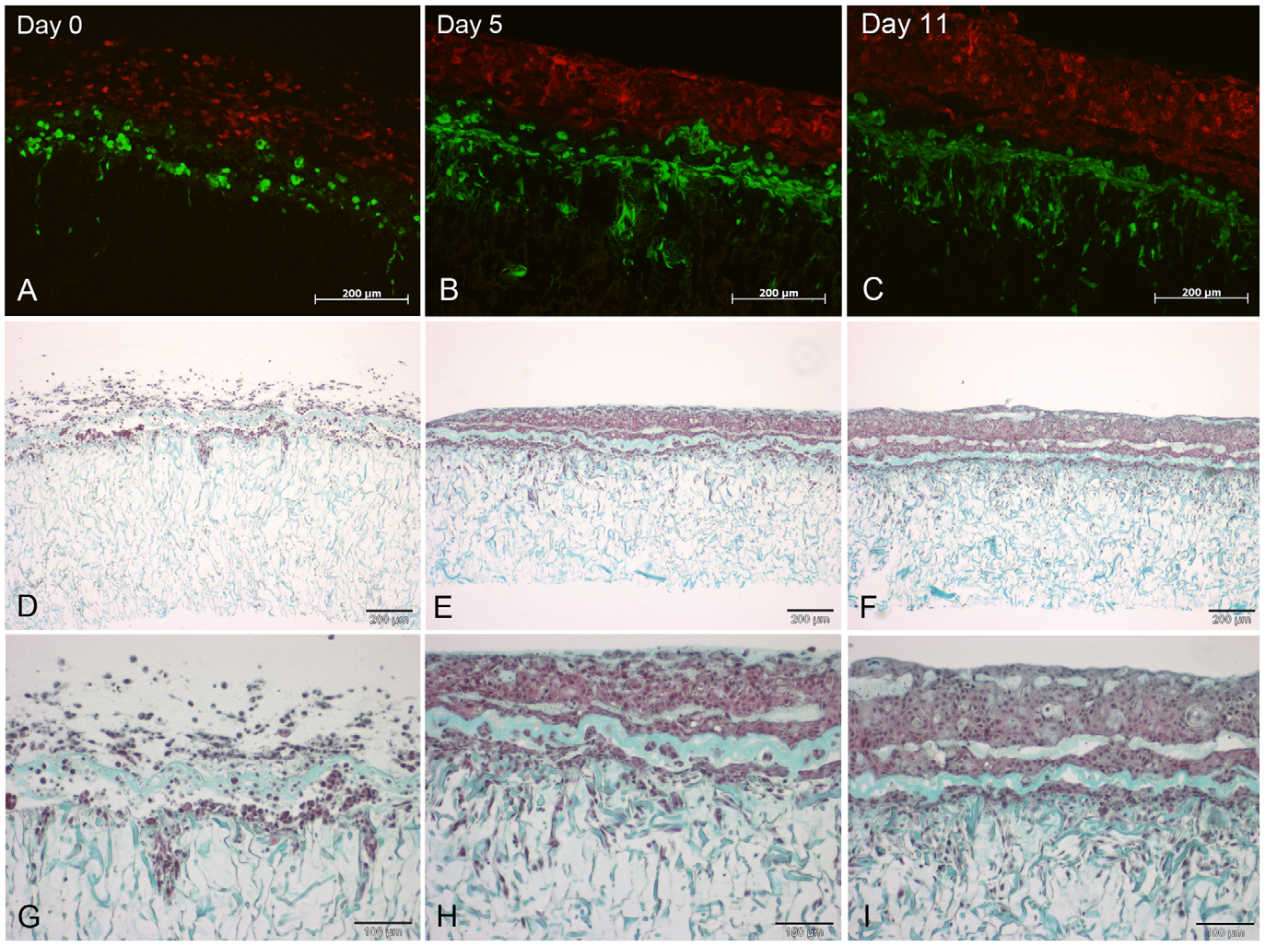
Histological sections of the tissue engineered skin constructs *in vitro.* Skin constructs were cultivated at the air-liquid-interface with differentiation medium for 11 days. Sections show cells using fluorescent microscopy and Masson’s trichrome staining, respectively. The time points indicated in A–C are valid for the whole respective columns. The skin constructs were cultivated at the air-liquid-interface. The keratinocytes (HaCaT-mCherry) exhibit red fluorescence while the fibroblasts (NIH3T3-eGFP) appear in green (A–C). Masson’s trichrome staining reveals the connective tissue containing collagen (green) and the cells (reddish) (D–I). The fibroblasts already start to grow into the Matriderm® underneath one day after printing (A, D, G). The keratinocytes, which still are rounded and are not connected to each other on day 0 (A, D, G), already form a dense tissue on day 5 (B, E, H). The thereby formed epidermis increases in height until day 11 (C, F, I). Scale bars depict 200 µm (A–F) and 100 µm (G–I).

On day 0 the two multi-layers of different cell types on top of the Matriderm® could clearly be seen (Figure 8A, D, G). The keratinocytes were still round and embedded in the collagen gel without contact to each other and with quite large spacing between the cells. In contrast, the fibroblasts already began to stretch out and to migrate into the Matriderm®. While the keratinocytes formed a dense tissue during the course of time, part of the fibroblasts migrated into the Matriderm®, following the fibres closely (Figure 8, brown cells beside green Matriderm® fibres, especially clear in Figure 8H and I).

A thickening of the epidermis-like tissue formed by the keratinocytes could be observed comparing day 11 to day 5 (Figure 8). Surprisingly, trichrome staining revealed the presence of a horizontal line of collagen in the lower part of the epidermis-like tissue and some globular collagen accumulations in the upper part. These probably are the remnants of the collagen used for the printing process.

**Figure 9:**
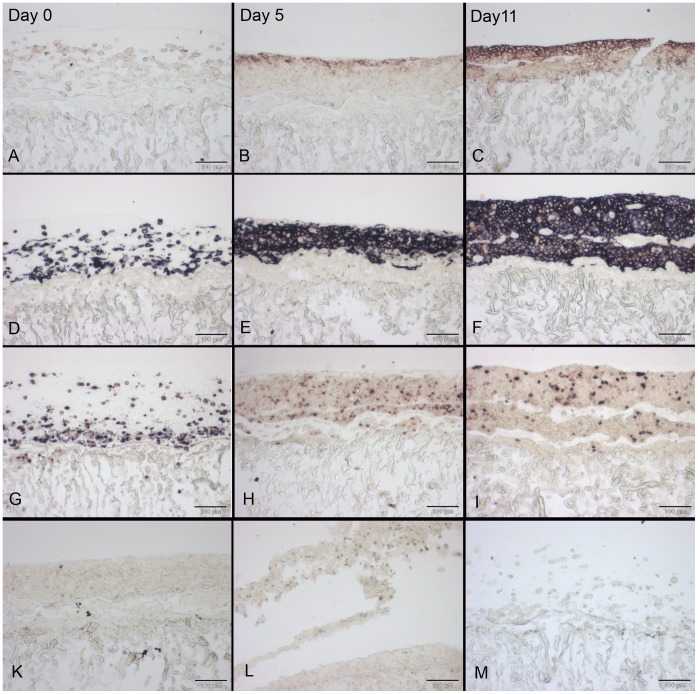
Sections of immunohistochemically stained skin constructs cultivated *in vitro*. Skin constructs were cultivated *in vitro* at the air-liquid-interface with differentiation medium for 11 days. The indicated time points in A–C are valid for the whole respective column. E-cadherin expression is absent on day 0 but can be detected on days 5 and 11 (A–C) while cytokeratin 14 expression is clearly visible at all time points in the whole epidermis (D–F). While nearly all cells exhibit Ki67 staining on day 0, only few cells do so at days 5 and 11 (G–I). The corresponding negative controls of the stainings (skin constructs without first antibody) are shown below (K – e-cadherin, L – cytokeratin 14, M – Ki67). All scale bars depict 100 µm.

Also in the *in vitro* cultures, immunostaining was carried out. The presence of e-cadherin increased during time from none on day 0 to its detection in the whole epidermis-like tissue on day 11, (Figure 9A–C). This is in accordance with the trichrome stainings, where a dense tissue can be observed on days 5 and 11 (Figure 8).

Also *in vitro,* cytokeratin 14 could be detected evenly distributed throughout all layers of the epidermis-like tissue (Figure 9D–F). This indicates a lack of differentiation. Proliferation could be found in all examined stages of the *in vitro* cultures (Figure 9G–I). While nearly all of the just printed cells showed proliferation on day 0, only some of the cells did so on days 5 and 11. But in contrast to the *in vivo* situation, the proliferating cells were found in the whole epidermis-like structure, more or less evenly distributed. No special spatial pattern could be observed.

## Discussion

The development of newly generated skin substitutes for burn therapy is very important. Here, we present the *in vivo* assessment of a simple skin equivalent created *via* LaBP. The printed cells form a tissue which is quite similar to native skin, including collagen producing fibroblasts and presumably differentiating keratinocytes, forming a dense epidermis. Although 11 days of cultivation is too short for a complete differentiation of keratinocytes, the distribution of Ki67 (as a marker for proliferation) mainly in the suprabasal layers hints at the beginning differentiation of the keratinocytes. In native skin, only the keratinocytes in the *stratum basale* maintain proliferation, whereas the differentiating keratinocytes in the other skin layers cease proliferating. Furthermore, the Matriderm® carrier becomes populated by the printed fibroblasts (presented in this work) as well as murine host fibroblasts (presented in previous work [20]). This leads to the integration and ingrowth of the skin construct into the wound. However, the absence of rete ridges and the thinner epidermis in the skin constructs may result in less stability of the constructs compared to native mouse skin. This may be solved by printing rete ridges and a thicker epidermis in future experiments, though.

As skin is a complex organ consisting of different cell types and substructures arranged in defined spatial patterns, LaBP is suited for the production of tissue engineered skin substitutes. It offers many possibilities and is a very promising technique for the fabrication of other kinds of tissues as e.g. bone or cartilage [8] [10]. [12]. Different levels of tissue generation have been investigated using bioprinting techniques. As we have previously shown *in vitro*, printed epidermal cells develop a dense epidermis including the expression of adherens and gap junctions [17]. In this manuscript, the next step has been carried out and tissue formation also *in vivo* could be documented. In a different setting, a pie-shaped multi-layered construct produced by inkjet printing (see below) and consisting of different cell types (stem cells, smooth muscle cells, endothelial cells) has already been analysed *in vivo* as a technical prerequisite to develop vascularised bone tissue in the future. The implanted cells could be detected several weeks after implantation indicating good survival rates. Interestingly, the used stem cells were able to differentiate into bone *in vivo* and endothelial cells formed a network of blood vessels in the implants after six weeks [21].

Concerning maintenance of cellular phenotype, human chondrocytes were found to express cartilage specific genes after being printed into cartilage lesions by inkjet printing and being cultivated *ex vivo*. They maintained their deposited positions due to simultaneous photopolymerization of a surrounding biomaterial scaffold, and attached firmly to the enclosing cartilage tissue [22]. These findings are in accordance with our own experiences [16] and that of others [7] [9] [13]–[14] as LaBP does not seem to impair cellular phenotype and behaviour. Even the differentiability of transferred mesenchymal stem cells [11]–[12] and pluripotent murine embryonal carcinoma cells [15] is maintained.

In the *in vitro* samples, a horizontal stripe of collagen without cells could be observed in the lower part of the epidermis-like structure. Obviously, the fibroblasts did not degrade the circumjacent collagen - which has been used as a hydrogel during the printing process - but started to migrate into the Matriderm® right away. During the printing, the cells are mixed with a hydrogel (in general called printing matrix) to serve four different purposes. Firstly, the matrix is necessary to achieve a uniform coverage of the donor glass slide with the biomaterial to be transferred. This is important as only then a consistency and uniformity of the printing process is possible. Secondly, the matrix helps the cells to survive, providing a moist environment preventing drying. Thirdly, it presents a specific surrounding for the printed cells and thereby acts as a biomimetic gel to create the desired micro-environment. In our case, collagen is already present in physiological skin and therefore is very suited as a printing matrix. Fourthly, a matrix like collagen enables the formation of a 3D construct due to its gelling effect. While in the *in vitro* controls the collagen is left by the fibroblasts, in the *in vivo* situation outstretched fibroblasts can be found in the collagen, probably also expressing and producing collagen by themselves. As this is similar to the situation in physiological skin, this process is much desired.

Very important for the take of a grafted skin or skin substitute is its fast vascularisation. It is a major prerequisite for the successful clinical use of a skin substitute. In our experiments, blood vessels could be found to start growing into the Matriderm® from the wound bed and the wound edge mostly in the direction of the transplanted cells. In a previous study, in skin constructs consisting of Matriderm® covered with collagen type I but without cells [20] no vessels could be detected growing into the Matriderm®. This suggests that the neovascularisation might be induced or supported by the printed cells on top of the Matriderm®. Actually, keratinocytes were found to produce vascular endothelium growth factor (VEGF) [23], the expression of which is regulated by several growth factors and cytokines [24] as well as by insulin in a diabetic mouse model [25]. Furthermore, keratinocytes in a tissue engineered skin substitute regulate the size of newly growing vessels in the dermis *in vitro*, resulting in small vessels similar to capillaries present in the skin’s microcirculation [26]. The same effect could be observed in the absence of keratinocytes when adding keratinocyte-conditioned medium or VEGF. Therefore, we assume that the printed keratinocytes in our skin substitutes might enhance vessel formation by VEGF production.

In our experiments no complete vascularisation of the printed skin equivalents could be achieved under the current conditions. Probably, the period was too short for complete vascularisation of the skin equivalent. Following the idea of improving graft vascularisation, Black *et al.* integrated human umbilical vein endothelial cells (HUVEC) into tissue engineered skin equivalents containing fibroblasts and keratinocytes in combination with a collagen chitosan scaffold [5] [27]. According to their study, the HUVEC were shown to form capillary-like tubules in the dermis *in vitro.* In a similar approach, skin equivalents constructed by seeding acellular dermis with keratinocytes and Bcl-2-transduced HUVEC showed perfusion through HUVEC-lined microvessels two weeks after implantation into mice [28]. This highlights the necessity but also the probable success to incorporate endothelial cells into our printed constructs as a next step.

In contrast to the *in vivo* situation, our *in vitro* controls formed a multi-layered tissue with collagen producing fibroblasts, but did not show any differentiation of the keratinocytes (HaCaT). This might be due to the culturing method *in vitro*. Although differentiation aiding supplements were added to the culture medium and the skin constructs were raised to the air-liquid-interface, this might not have been appropriate enough to trigger the differentiation. Also, the culturing period of 11 days is quite short and induction of differentiation *in vitro* might have been observed at a later time point. However, the beginning differentiation of the keratinocytes *in vivo* could be due to the growth factors present in mice but absent *in vitro*.

Summing up, LaBP offers the possibility to place cells of a specific type wherever in the tissue they are needed. This is a unique feature of bioprinting techniques and it may be used to print skin supplemented with endothelial cells, hair follicle cells, peripheral nerve cells, Schwann cells, melanocytes or cells present in perspiratory and sebaceous glands. Therefore, we hope to be able to produce a much more similar skin construct to native skin compared to other current approaches. This is especially important for the future patients as they gain a much more functional and aesthetic skin substitute. This in turn would lead to a major increase of their quality of life, on the physical as well as on the mental level.

As an alternative to LaBP, a similar technique called inkjet printing is available, which has already been used to print 2D protein arrays [29], endothelial cells, smooth muscle cells [29]–[30], a 3D construct containing HeLa cells [31], or a 3D composite construct containing muscle cells, endothelial cells and stem cells [21]. Also, inkjet printing can be used to create antimicrobial assays [32] or to transfect cells with relatively large molecules [33]. The major disadvantage of this technique, however, is the high shear force of the nozzle, leading to severe cell impairment [34]. With LaBP – which is nozzle free – cells can be printed with a much higher density [10]. This is very important for the printing of skin, which is a tissue with a very high density of cells present. Therefore, for our purposes, LaBP remains the technique of choice.

To further improve the use of LaBP for the creation of (skin) tissue, an adaptation to automation would be of advantage. Our setup is not suited for the high throughput production of skin substitutes yet, but in principal an automation of the whole process - including the printing process as such as well as the cultivation of the skin substitutes - is conceivable.

We used the dorsal skin fold chamber in mice for the assessment of the tissue engineered skin constructs. This approach exhibits different advantages as well as drawbacks. The common approach of dorso-lateral full-thickness wounds without a chamber allows for the cultivation of a tissue engineered skin constructs for a long period of time. While two to eight weeks are the most frequently used time intervals [35]–[38], animals can be kept up to six months [39]. Using the chambers and the small nude mice, the observation period is quite limited as the mice would not be able to bear the weight of the chambers for several weeks. In our study, we demonstrated that skin constructs produced by LaBP are viable *in vivo*, forming a tissue similar to simple skin within the time frame of 11 days. As a further limitation, compared to the common approach of simple full-thickness wounds in the dorso-lateral region of mice with an area of 2 cm×2 cm to 2 cm×3 cm [35] [38]–[40], the wound area in the chambers is very small (round hole with 6 mm diameter). This, however, is partly compensated by the lack of wound contraction. The latter is the major way of wound healing in rodents [41], opposed to the main mechanisms in human wound healing, i.e. granulation tissue formation and subsequent reepithelialisation [3] [42]. In the chambers, the skin constructs are safely secured in the wound by the glass slide while the surrounding and opposite skin is firmly attached to the titanium frames [20]. Therefore, no contraction of the wound area occurs. This is very important since we aim at assessing the situation in humans and not in rodents. A further advantage of the skin fold chamber is the lack of customary wound dressings. Thereby, no changes of the dressings are necessary, which reduces the stress for the animals considerably [20]. Furthermore, the transparent glass slide allows for a continuous observation of the wound closure, without any stress for the animals due to the removal of wound dressings [43].

In conclusion, we could show LaBP to be an adequate technique for the creation and *in vivo* formation of a 3D tissue like skin. Therefore, LaBP represents a major promise for the improvement of burn therapy and thus for a raised quality of life for the patients.
